# Computational detection and suppression of sequence-specific off-target phenotypes from whole genome RNAi screens

**DOI:** 10.1093/nar/gku306

**Published:** 2014-06-27

**Authors:** Rui Zhong, Jimi Kim, Hyun Seok Kim, Minsoo Kim, Lawrence Lum, Beth Levine, Guanghua Xiao, Michael A. White, Yang Xie

**Affiliations:** 1 Quantitative Biomedical Research Center, Dallas, TX, USA; 2 Department of Clinical Sciences, Dallas, TX, USA; 3 Department of Cell Biology, Dallas, TX, USA; 4 Severance Biomedical Science Institute, Yonsei University College of Medicine, Seoul, Korea; 5 Simmons Comprehensive Cancer Center, Dallas, TX, USA; 6 Center for Autophagy Research, Dallas, TX, USA; 7 Howard Hughes Medical Institute, UT Southwestern Medical Center, Dallas, TX, USA

## Abstract

A challenge for large-scale siRNA loss-of-function studies is the biological pleiotropy resulting from multiple modes of action of siRNA reagents. A major confounding feature of these reagents is the microRNA-like translational quelling resulting from short regions of oligonucleotide complementarity to many different messenger RNAs. We developed a computational approach, deconvolution analysis of RNAi screening data, for automated quantitation of off-target effects in RNAi screening data sets. Substantial reduction of off-target rates was experimentally validated in five distinct biological screens across different genome-wide siRNA libraries. A public-access graphical-user-interface has been constructed to facilitate application of this algorithm.

## INTRODUCTION

Genome-wide high-throughput RNA interference (RNAi) screening has been widely applied in biomedical research for discovery of drug targets or illumination of unknown molecular machinery, and has proven to be an effective means for functional annotation of protein-coding genes in both normal and disease contexts (1–5). However, a pressing challenge for these studies is maximizing the return of accurate gene-level information with a technique that is associated with pleiotropic mechanisms of action. For example, multiple studies indicate that individual small interfering RNAs (siRNAs) often interfere with the expression of hundreds of genes through partial sequence complementarity that imitates microRNA (miRNA) activity (6,7). Therefore, the phenotypic read-outs from siRNA screens are usually comprised of both the desired ‘on-target’ effects of intended target gene depletion together with unintentional ‘off-target’ effects that are oligonucleotide sequence dependent, but target gene-independent. The latter can lead to many false positive ‘hits’ that subsequently obscure interpretation of the overarching screen results. Time- and resource-intensive experimental approaches for target validation therefore often define the limits of the reliable gene-level information from any given screen. Computational approaches have been designed which can help identify off-targeted transcripts within a given screening effort, and therefore lead to the discovery of new genes or pathways associated with the phenotype under investigation (8,9). However, directly addressing high false positive rates and deconvolution of off-target phenomena is still a major bottleneck restraining the pace of discovery for functional genomics efforts. To address this issue, we developed a computational approach, Deconvolution Analysis of RNAi screening data (DecoRNAi), for automated quantitation of off-target effects in RNAi screening data sets.

## MATERIALS AND METHODS

### Data processing

DecoRNAi approach has been tested in five distinct biological screens across different genome-wide siRNA libraries, and all data processing and *Z* score derivations were consistent with the original publications (1–5).

1. For the H1155 toxicity screens (1), host modulators of H1N1-cytopathogenicity (3) and the HCC4017 toxicity screens (5), raw cell viability data were transformed to robust *Z* score (formula shown below) and adjusted for batch effect. That is, raw data were grouped by experimental batch and within each group, sample median and median absolute deviation were used to calculate robust *Z* score. Annotation of all siRNA/miRNAs pools and their associated *Z* scores can be found in Supplementary Tables S1, S2, S3 and S6.
  \begin{equation*} Z = \frac{{{\rm cell}\,{\rm viability} - {\rm sample}\;{\rm median}}}{{{\rm median}\;{\rm absolute}\;{\rm deviation}\;({\rm MAD})}} \end{equation*}
  \begin{equation*} {\rm MAD} = {\rm median}_i \left( {|X_i - {\rm sample}\;{\rm median}|} \right) \end{equation*}
2. For the WNT (int/Wingless) pathway siRNA screen (4), *Z* scores were calculated as a standard score centered on the population mean of each screening run as described by the average of each triplicate experiment minus the standard deviation. Annotation of all siRNA pools and their associated *Z* scores can be found in Supplementary Table S4.
3. For the selective autophagy siRNA screen (2), mitochondrial mass for each cell was approximated by the following formula: mitochondrial mass ∼ *β*_0_ + *β*_1_ Parkin + *β*_2_ siRNA + β_3_ Parkin × siRNA. Two-way ANOVA models were used to identify siRNAs that decreased Parkin-mediated mitophagy and *Z* scores were calculated as the statistical significance. Annotation of all siRNA pools and their associated *Z* scores can be found in Supplementary Table S5.

### DecoRNAi analysis

The LASSO (least absolute shrinkage and selection operator) regression approach was adapted to quantify the strength of seed-link effects. For this analysis, each *Z* score is modeled as a linear combination of on-target effect (demonstrated in the Supplementary Figure S5) and seed sequence based off-target effects. The LASSO regression model was defined as below:

\begin{equation*}
\vec Z = X\vec \beta + \vec Y,\;{\rm subject}\;{\rm to}\;|\vec \beta | < s
\end{equation*}

where *Z_i_* is the *i*th original *Z* score, *β_j_* is the estimated off-target effect of the *j*th seed family, *Y_i_* is the corrected *Z* score (on-target effect) and *λ* is the penalty parameter. *X* is denoted as below:

\begin{equation*}
X = \left[ {x_{ij} } \right],\;x_{ij} = \left\{ {\begin{array}{*{20}c} {1,\quad {\rm if}\quad {i} \in {j}{{\rm th}} \;{\rm family}} \\
{0,\quad {\rm otherwise}} \\
\end{array}} \right. 
\end{equation*}

And the solution is given:

\begin{equation*} \hat \beta = \mathop {\arg \min }\limits_\beta \left[ {||\vec Z - X\vec \beta ||^2 + \lambda |\vec \beta |} \right] \end{equation*}

For each seed family, we can thus estimate the coefficient that indicates the strength and direction of predicted off-target effects. A negative coefficient means the seed family tends to lower *Z* scores and vice versa. Based on empirical experience, *λ* is set to 0.001 as the default. We annotate those coefficients with absolute value >*1* as indicating candidate off-target effects for all four datasets shown in this manuscript. However, all the parameters and cutoff values are tunable by users.

For LASSO-selected off-target seed families, we further examine the statistical significance using the Kolmogorov-Smirnov test (KS-test). Taking $\vec Z$ as a vector of original *Z* scores from primary screening, the empirical distribution function *F_n_* for *Z* scores from seed family *S* is defined as:

\begin{equation*} F_{n_s } (z) = \frac{1}{{N_{\rm s}}}\sum\limits_{i = 1}^{N_{\rm s} } {I_{Z_i \le z} } \end{equation*}

where $I_{Z_i \le z}$ is the indicator function, equal to 1 if $Z_i \le z$ and equal to 0 otherwise, *N*_s_ is the total number of *Z* scores from seed family S. The KS statistic for a given cumulative distribution function *F*(*z*) is as:

\begin{equation*} D_{n_s } = \mathop {\sup }\limits_z |F_{n_s } (z) - F(z)| \end{equation*}

The statistical significance (*P*-value) was then determined by the KS statistic.

### **Web-base**d application (Galaxy)

The DecoRNAi application is available at http://galaxy.qbrc.org/root?tool_id=sirna_offtarget, which is an open web-based interface. Analysis parameters can be specified by users as below:

- InputFile: CSV File containing response variable and siRNA sequence data.
- Strand: Specify the strand orientation for analysis.
- Lambda: Penalty parameter used in the model.
- Seed: Range: 1–14. Specify the seed region to be used.
- Library: Specify siRNA library. Default is custom which requires user input sequences.
- Strength: Specify the cutoff for strength of seed-linked effect. Must be positive value.
- Significance: Specify the cutoff for significance (*P*-value).

### Tissue culture, oligo transfection and cell viability assays

In primary screening, all projects employed pooled siRNAs targeting strategy. The sequences of pooled siRNAs are different. However, by design, the siRNAs within the same pool should be targeting the same gene by perfectly matching on different location of the corresponding messenger RNAs (mRNAs) (Supplementary Figure S5).

In secondary individual oligo screening, H1155 cells were grown in RPMI 1640 (Gibco^®^) supplemented with 5% fetal bovine serum (FBS; Atlanta Biologicals) and 1% penicillin/streptomycin (Gibco^®^). All siRNAs were purchased from Dharmacon. The library contains 24 sets of four siRNAs each. The oligos targeting transmembrane protein 114 (TMEM114) from Dharmacon were used for siRNA negative control. The miR 4633-5p and the synthetic miRNA were from Ambion. Nontargeting miRNA control (IN-001005-01-05) was from Dharmacon. For reverse transfection, 1 μl siRNA (10uM) in 30 μl serum free media (SFM) was mixed with 0.4 μl RNAi Max (Invitrogen) in 10 μl SFM. 40 μl siRNA-reagent mix per well and 5000 cells per well, from a single cell suspension, were delivered in 100 μl media in 96-well microtiter plates. Cell viability was measured 96 h post-transfection with CellTiter-Glo (Promega) according to manufacturer's specifications.

## RESULTS

**Figure 1.**
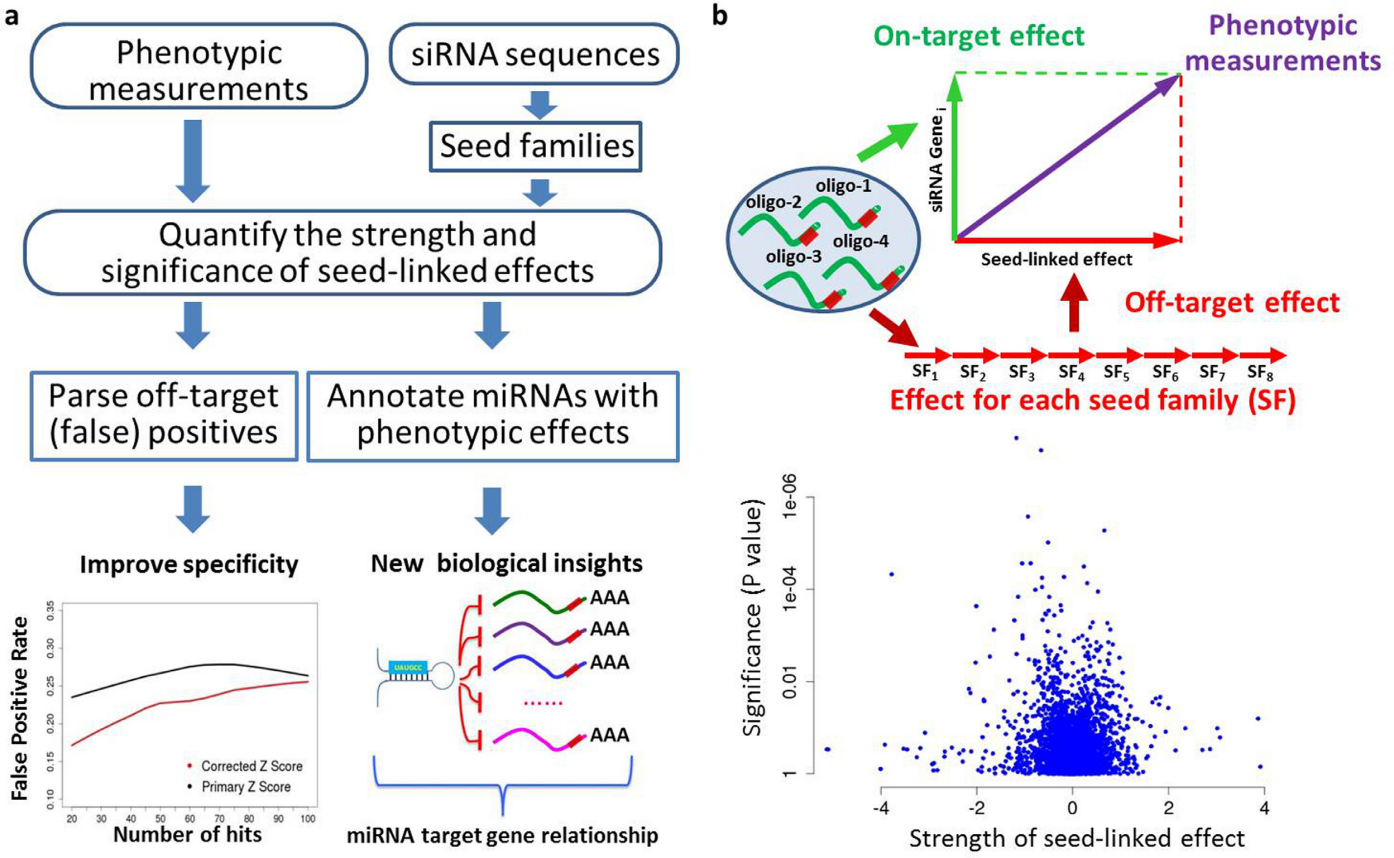
Deconvolution analysis of RNAi screening data (DecoRNAi). (**a**) Workflow of DecoRNAi analysis. (**b**) Original phenotypic measurements (*Z* scores, etc.) are projected on both on-target effect (green) and off-target effect (red) in a deconvolution pattern (top panel). A penalized linear model is used to quantify on-target and off-target effects. The resulting seed family scores from a genome-wide H1155 siRNA toxicity screen are shown (bottom panel). Each dot represents a seed family including all siRNAs sharing the same seed sequence. X-axis is estimated strength of seed-linked effect (off-target effect) and Y-axis is *P* value associated with each seed family.

Here we have designed a data-driven computational approach, DecoRNAi, to quantify and correct miRNA-mimic off-target effects from whole-genome RNAi screens (Figure 1a). DecoRNAi simultaneously estimates gene-level on-target effects and siRNA oligonucleotide-level off-targets effects based on deconvolution of phenotypic measurements from primary screening data (Figure 1b). Experimental evaluation, using thousands of oligonucleotide retests across five independent whole-genome siRNA screens, indicated miRNA mimicry by siRNA oligonucleotides is a pervasive source of ‘off-target’ biological responses to siRNA. Application of DecoRNAi significantly enhanced the fidelity of single gene-level observations at whole-genome scale, and is provided here an open-source tool to enhance lead discovery accuracy from high-throughput RNAi screening studies.

A major determinant of the mRNA targets for translational suppression by a given miRNA, is partial mRNA/miRNA complementarity corresponding to a 6-nucleotide ‘seed sequence’ on the 5′ end of the miRNA (10). By this definition, there is a sum total of 4^6^ (4096) possible non redundant ‘seed sequences’ within any siRNA or small hairpin RNA (shRNA) library collection. As there are usually tens to hundreds of thousands of oligos in a given screening collection, the presence of a given seed sequence within many different siRNA/shRNA reagents presents the opportunity to identify ‘seed-driven’ phenotypic associations among reagents within a given screen. To begin to test this, we examined a whole-genome siRNA toxicity screen in H1155 non-small cell lung cancer cells (3) (Supplementary Table S1) as a benchmark for identification of a reasonable scoring approach. This is a cell-based high-throughput RNAi screen to identify genes required for lung cancer cell viability. This screen employed an arrayed one-gene/one-well commercial siRNA library with pools of four independent siRNA oligonucleotide duplexes per gene. Seed sequence membership for each of the 168 992 oligonucleotides in the library was separately defined for each of the 6-mer windows present within each 19-mer (Supplementary Figure S1a). The toxicity value for each siRNA pool was calculated as a *Z* score from a triplicate analysis in order to control for position and batch effects (11). We employed a two-sample KS-test as a nonparametric method to discover oligonucleotide-specific effects based on the coherent behavior of groups of siRNA pools with oligos sharing a common seed sequence (Supplementary Figure S1b). Using a confidence threshold of a false discovery rate (FDR) of 5%, seed family/phenotype associations were detected only within seed families defined by positions 1–6, 2–7 and 3–8 (Supplementary Figure S1c). The majority corresponded to a seed definition of nucleotides 2–7, in keeping with current best estimations of dominant determinants of miRNA target specificity (10). Using that definition for seed family membership, there are 4001 unique seed sequences represented among the 168 992 Dharmacon oligonucleotides, with a median frequency of representation of 27 siRNA pools (Supplementary Figure S1d). A liability of the KS-test is sensitivity to family size. This can result in the discovery of associations with very low *P*-values but very small effect sizes (Supplementary Figure S1e–g). To defend against that, we developed DecoRNAi algorithm that estimated strength and direction of seed-driven effects using LASSO as a penalized regression model (see ‘Materials and Methods’ section).

**Figure 2.**
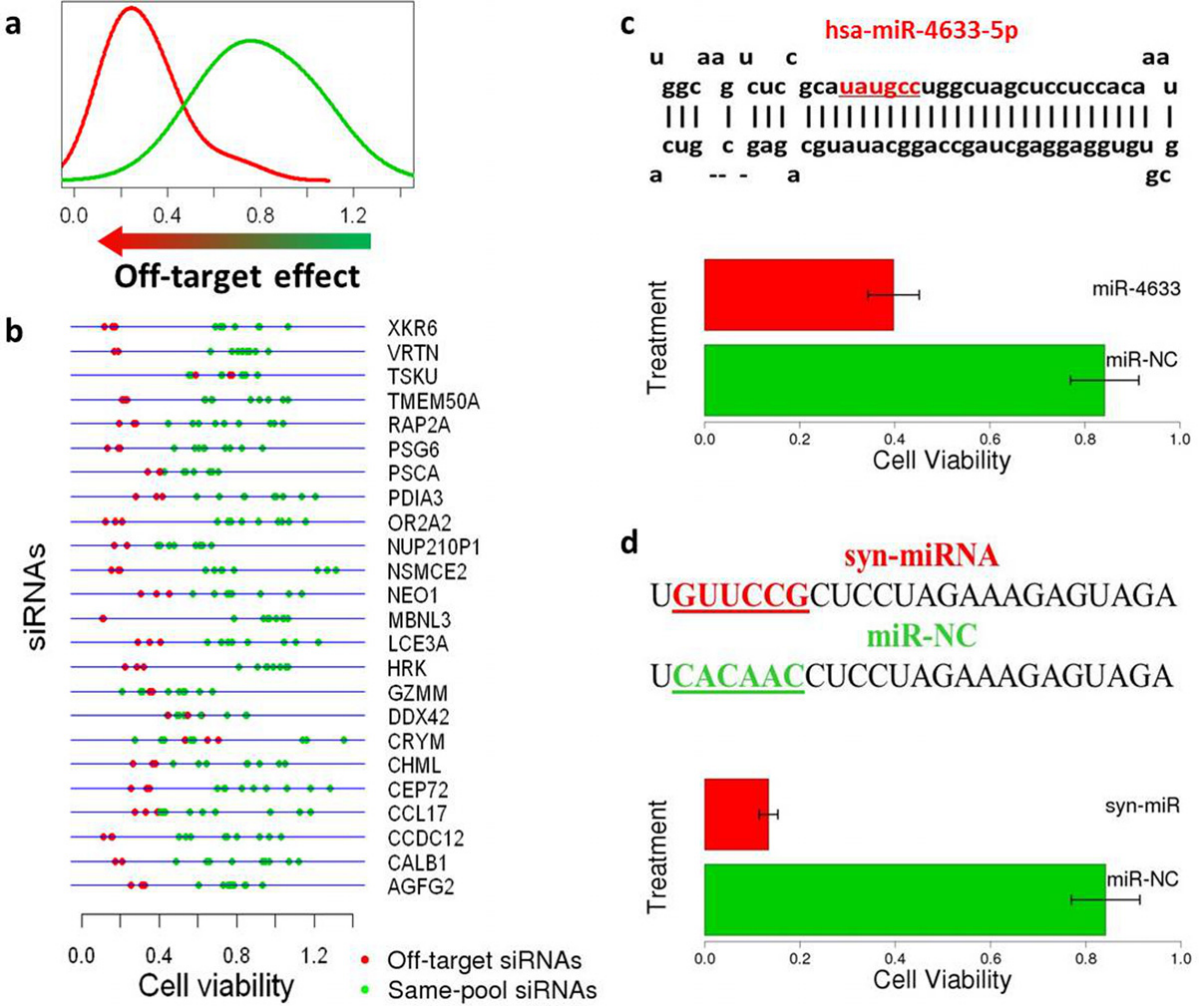
Experimental evaluation of seed-effect predictions. (**a**) Density distributions of consequences on cell viability (X-axis) of siRNA duplexes containing predicted off-target seed sequences (red curve) versus siRNA duplexes targeting the same genes but without predicted off-target seed sequences (green curve). (**b**) Cell viability in response to individual siRNA oligo duplexes from 24 gene pools tested in H1155 cells. Red dots indicate duplexes with an oligonucleotide containing the candidate off-target seed sequence. Green dots represent siRNA duplexes, designed to target the same gene, which do not contain the candidate off-target seed sequence. (**c**) An oligonucleotide mimic of hsa-miR-4633 significantly inhibited H1155 cell viability. Hsa-miR-4633 contains the seed sequence UAUGCC that was identified as an off-target seed through DecoRNAi analysis. (**d**) A synthetic miRNA mimic designed using the predicted off-target seed GUUCCG significantly inhibited H1155 cell viability, while a control miRNA mimic of identical sequence with the exception of the indicated seed region did not.

Application of DecoRNAi to the H1155 toxicity screen identified 13 potential seed-driven phenotypic associations corresponding to 365 siRNA pools (Supplementary Figure S2a). For experimental evaluation of candidate seed-driven effects, we chose four ‘off-target’ seed families (GUUCCG, UCCAGG, UUGCAG, UAUGCC) for a focused secondary screen (Supplementary Figure S2c–f). Twenty-four genes were selected and for each gene, the corresponding Dharmacon siRNA pool was retested as four individual siRNAs for consequences on H1155 cell viability. Of note, siRNA duplexes containing an oligo with the detected off-target seed sequence were consistently associated with stronger consequences on cell viability than the remaining siRNA duplexes designed to target the same gene (Figure 2a, b, Supplementary Figure S2g). Consistent with this, the cumulative density function indicates a significant shift in the *Z* scores associated with predicted seed-driven off-target effects (Supplementary Figure S2b, *P* = 4.56 × 10^−13^).

Of the detected seed families, only one (UAUGCC) is present within the seed sequence of an annotated human miRNA (hsa-miR-4633-5p). As expected, introduction of the miRNA mimic corresponding to miR-4633-5p resulted in a similar viability defect (Figure 2c). To further test the sufficiency of seed sequence identity for induction of off-target phenotypes, we engineered a synthetic miRNA corresponding to the validated seed family GUUCCG (Supplemental Figure S2c, g). This reagent also effectively inhibited viability of H1155 cells, consistent with a dominant seed-sequence dependent mode of action (Figure 2d).

We next applied DecoRNAi to four additional whole-genome siRNA screens employing distinct biological contexts and endpoint assays. These included a siRNA and miRNA mimic screen for host modulators of H1N1-cytopathogenicity, a siRNA screen for modulators of WNT reporter gene activation, a siRNA image-based screen for selective autophagy factors and one additional screen for lung cancer drug target discovery using a distinct whole-genome siRNA library.

**Figure 3.**
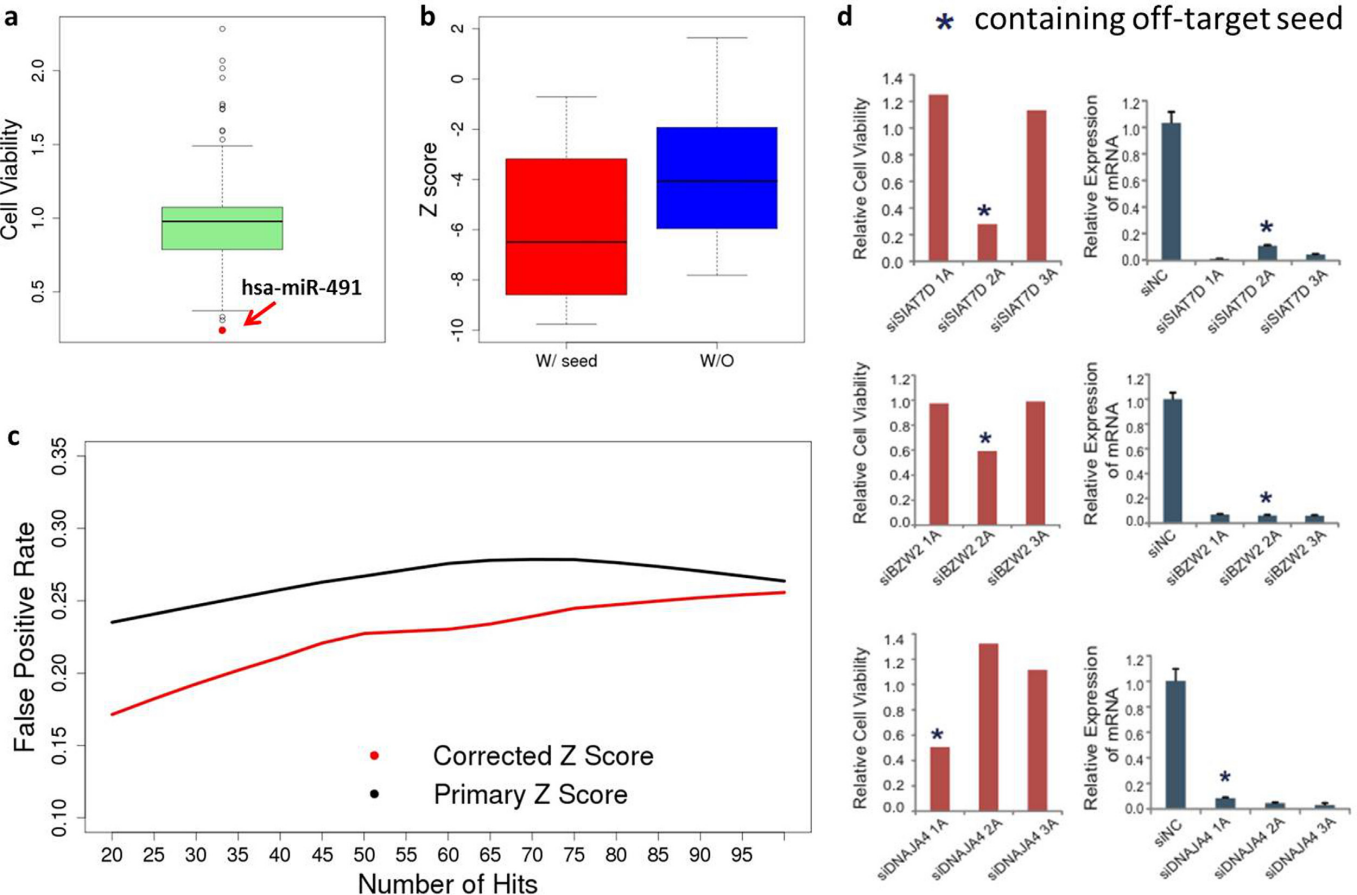
Application of DecoRNAi to additional RNAi screens with distinct biological contexts. (**a**) Identification and estimation of seed-sequence dependent off-target effects from a genome-wide siRNA screen for modulators of H1N1 induced cell death in HBEC30 cells. Consequences of 426 MicroRNA mimics on HBEC30 cell viability upon H1N1 infection are shown. hsa-miR-491, which contains a predicted off-target seed sequence, is as indicated. Out of all miRNA mimics, hsa-miR-491 has the lowest cell viability. Annotation of all miRNA mimics and their associated viability scores can be found in Supplemental Table S3. (**b**) Identification and estimation of seed-sequence dependent effects from a genome-wide siRNA screen for modulators of selective autophagy in HeLa cells. Phenotypic *Z* scores from secondary screens of individual siRNA duplexes with and without predicted off-target seed sequences are plotted as indicated. (**c**) DecoRNAi-mediated *Z* score corrections reduce false positive rates in autophagy modulator screen. Here, gene targets scoring positive with two or more confirmed siRNAs out of a total of four are considered to be true positives. The X-axis indicates arbitrary ‘hit’ thresholds based on rank-ordered *Z* scores and the Y-axis indicates the corresponding false positive rate. For example, the false positive rate for the top 20 ‘hits’ rank ordered by *Z* score is 24% when using the primary *Z* score and 17% when using the corrected *Z* score. (**d**) Identification and estimation of seed-sequence dependent off-target effect from a genome-wide siRNA toxicity screen on HCC4017 using an alternative distinct siRNA library. Individual oligos from three genes in HCC4017 screen data were tested as indicated. Viability phenotypes were uncoupled with from target gene knock-down. Individual oligos from three genes in HCC4017 screen data were tested. Target genes were knocked down by all siRNAs, however, only oligos with off-target seeds reduced cell viability.

The H1N1-cytopathogenicity screen sought to return genes that modulate influenza virus replication in human bronchial epithelial cells (3) (Supplementary Tables S2 and S3). For the primary screen, synthetic interactions of siRNAs and miRNA mimics with H1N1-induced cytopathogenicity were measured using cell viability as the endpoint assay. From the siRNA screen, DecoRNAi identified 13 significant seed families corresponding to eight synthetic lethal associations (353 siRNA pools) and five synthetic viable associations (96 siRNA pools) (Supplementary Figure S3a). One of eight synthetic lethal associations corresponded to a human miRNA; hsa-miR-491. Of note, the hsa-miR-491 mimic was also the best scoring synthetic lethal reagent from the miRNA mimic screen (Figure 3a).

The WNT screen sought to return genes modulating WNT pathway activation, and thus employed a highly specific endpoint assay using a WNT-specific and a WNT-independent reporter gene combination (4) (Supplementary Table S4). Here, only one seed-sequence association was identified among reagents that selectively inhibited WNT reported activity (Supplementary Figure S3b). This may be indicative of the narrower biological space that can intersect the endpoint assay employed in this screening effort, and which is therefore less exposed to perturbation by multiple seed-family oligonucleotides.

The autophagy screen is an image-based screen, at single cell resolution, that sought to identify gene products required for virophagy by measuring colocalization of Sinbis virus capsid protein with autophagolysosomes (2) (Supplementary Table S5). DecoRNAi detected six significant seed-sequence associations with inhibition of selective autophagy corresponding to 125 siRNA pools (Supplementary Figure S3c). From 28 individual siRNA oligo retests, those belonging to the detected off-target seed families trended towards lower *Z* scores than those designed to target same genes but not belonging to the predicted off-target seed families (Figure 3b). Importantly, substitution of the original *Z* scores with DecoRNAi-corrected *Z* scores, which remove the estimated miRNA-like off-target effects from the overall phenotypic measures (‘Materials and Methods’ section), significantly reduced the experimentally determined false positive rate (Figure 3c).

**Figure 4.**
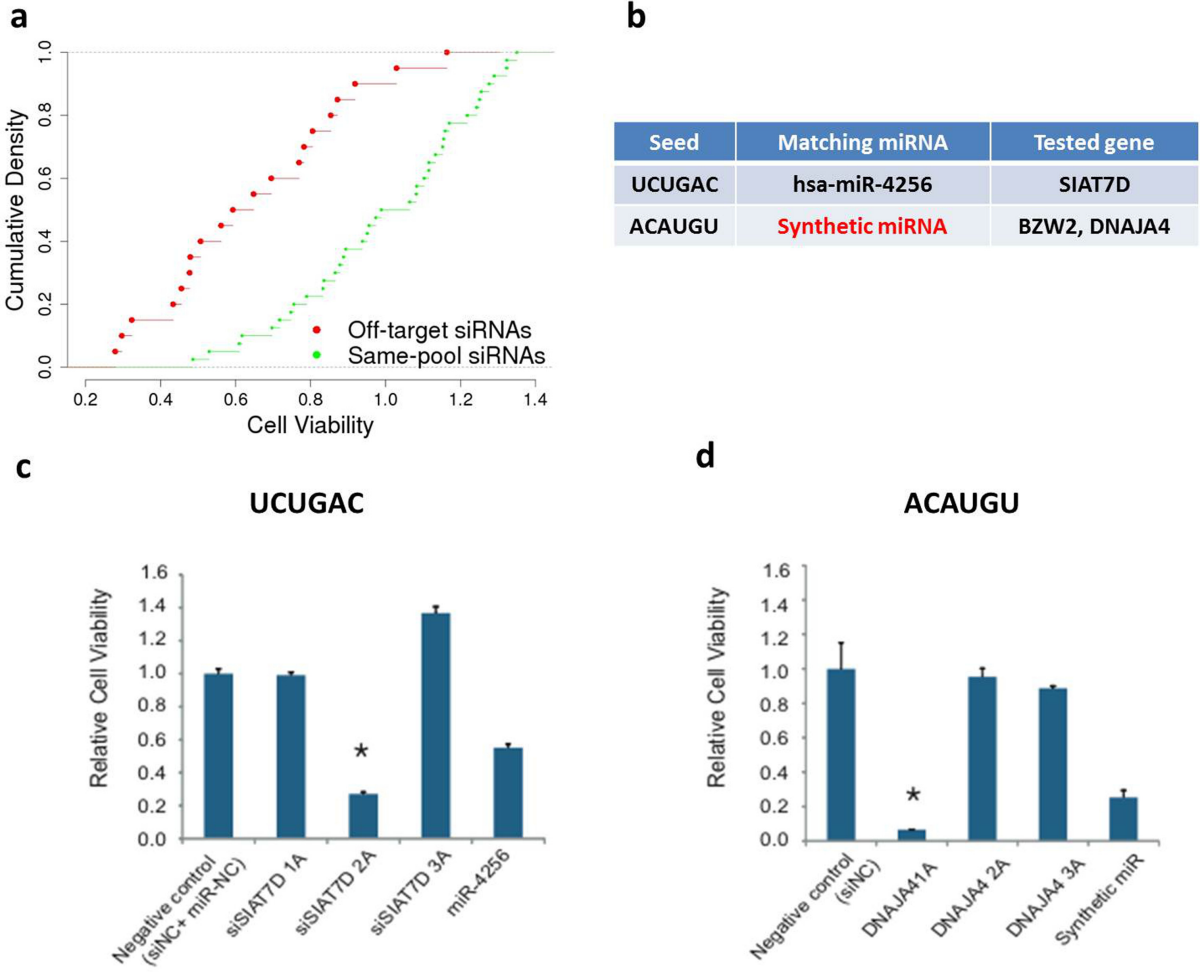
Application of DecoRNAi to a distinct siRNA library. (**a**) Identification and estimation of seed-sequence dependent effects from a genome-wide siRNA toxicity screen in HCC4017 cells using an alternative siRNA library. Density distributions of consequences on cell viability (X-axis) of siRNA duplexes containing predicted off-target seed sequences (red curve) versus siRNA duplexes targeting the same genes but without predicted off-target seed sequences (green curve). (**b**) Identified off-target seed UCUGAC and ACAUGU were retested using individual oligos designed to target SIAT7D, BZW2 and DNAJA4. miRNA mimic and synthetic miRNA were also retested. (**c**) An oligonucleotide mimic of hsa-miR-4256 sharing seed UCUGAC significantly inhibited HCC4017 cell viability. (**d**) A synthetic miRNA mimic designed using the predicted off-target seed ACAUGU significantly inhibited HCC4017 cell viability, while a control miRNA mimic of identical sequence with the exception of the indicated seed region did not.

To evaluate the performance of DecoRNAi using different genome-wide siRNA libraries, we examined additional toxicity screens designed to identify genes required for lung cancer cell viability (5). The non-small cell lung cancer cell line HCC4017 was screened for siRNA pools from the Ambion ‘Silencer-Select’ library that significantly impaired viability (Supplementary Table S6). DecoRNAi identified 10 off-target seeds from the library enriched in these screens (Supplementary Figure S3d). Testing of 60 individual siRNA oligonucleotides consistently showed that siRNAs containing these ‘off-target’ seeds have dramatic consequences on cell viability as compared to other siRNAs targeting the same genes (Figure 4a). For individual siRNA duplexes targeting SIAT7D, BZW2 and DNAJA4 (Figure 4b), mRNA expression data showed that all reagents successfully depleted the corresponding target gene mRNA, however, only oligos containing the predicted off-target seed had significant viability phenotypes (Figure 3d). miRNA and synthetic miRNA phenotypes were consistent with a dominant seed-sequence dependent mode of action (Figure 4b–d).

**Figure 5.**
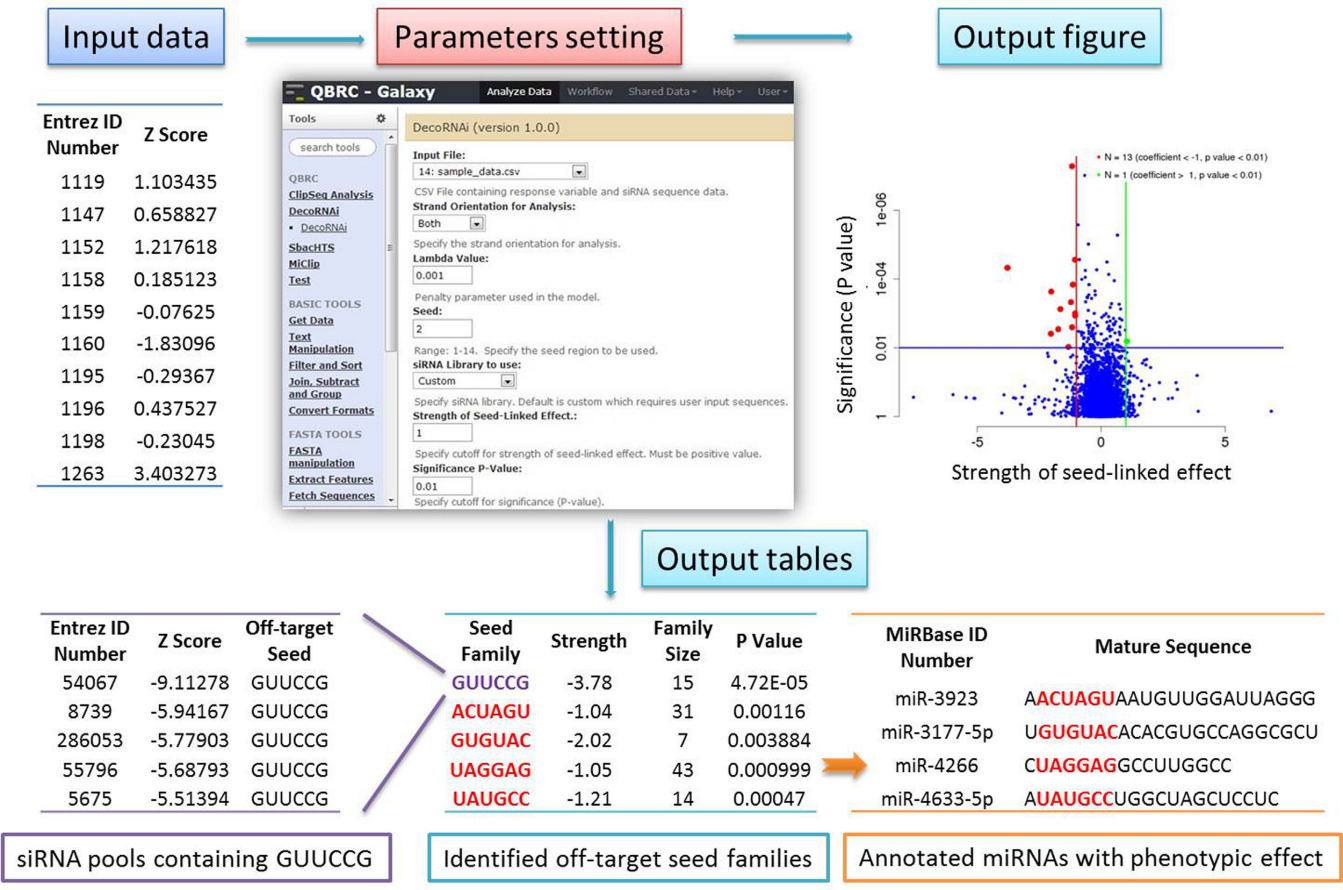
Illustrations of the web-based graphical user interface for DecoRNAi analysis. Seed families are pre-computed for the Dharmacon Library circa 2005, Dharmacon Library circa 2009 and Ambion Silencer Select. For screens employing these reagents, the only required input is the quantitative screen measurement for each reagent (for example, normalized *Z* score). Other libraries can be analyzed upon uploading the library-wide sequence information for each oligonucleotide or processed shRNA. Parameter settings are user-selected. The output files include global visualization of seed family behavior, the predicted off-target seed families, the siRNA pools containing off-target seed families, the potential miRNAs sharing common seeds with identified off-target seed families and the corrected *Z* scores (not shown here).

To facilitate application of the DecoRNAi algorithm, we have released a public access web-based graphical user interface http://galaxy.qbrc.org/root?tool_id=sirna_offtarget for custom analysis (Figure 5). This tool contains pre-computed seed sequence families for three commonly employed commercial siRNA libraries. For custom collections, the tool will compute seed sequence membership from a user-supplied reagent sequence table. The default parameters were provided for the DecoRNAi online tools based on the empirical performance, but all the parameters were tunable by users. The output files include global data visualization, the identified seed family associations, the siRNA pools containing off-target seed families, corrected *Z* scores and the potential miRNAs with phenotypes of interest (Figure 5, Supplementary User's Manual). To facilitate local software installation, we also developed an R package for user to perform custom analysis available on our galaxy webpage http://galaxy.qbrc.org/root?tool_id=sirna_offtarget.

## DISCUSSION

We constructed DecoRNAi to quantify seed-driven off-target activity by modeling the enrichment of oligonucleotide sequence-specific effects from genome-wide RNAi primary screen data. The approach does not require arbitrary phenotypic threshold selection, and combines statistical significance of population separation with phenotypic effect size to return biologically meaningful correlations. We have found that the algorithm performs well across diverse phenotypic assays and within distinct reagent collections. As expected, miRNA-like behavior of siRNA oligonucleotides was a pervasive feature associated with primary screening phenotypes. This was detectable by DecoRNAi, experimentally verifiable, and could be imitated with appropriately designed synthetic miRNA-like molecules.

**Figure 6.**
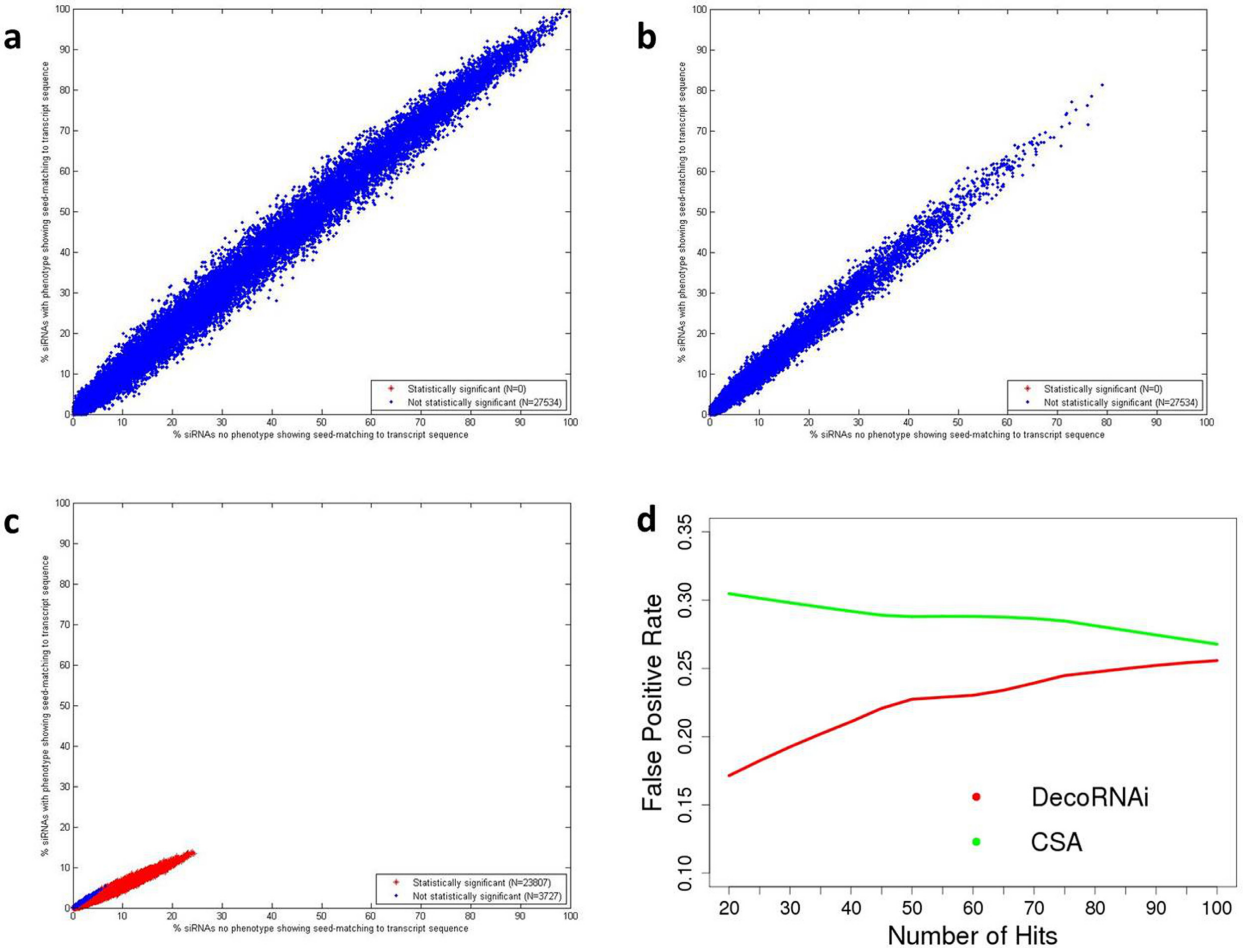
Comparison with GESS and CSA analysis. GESS analysis of human mRNA 3′ UTRs from primary data of the H1155 toxicity screen (**a**), the selective autophagy screen (**b**), and the H1N1 cytopathogencity screen (**c**). Each point represents one 3′ UTR and represents SMFa value plotted against the SMFi value. (**d**) DecoRNAi-mediated *Z* score corrections reduce false positive rates compared to CSA approach from selective autophagy screen. Here, gene targets scoring positive with two or more confirmed siRNAs out of a total of four are considered to be true positives. The X-axis indicates arbitrary ‘hit’ thresholds based on rank-ordered *Z* scores after applying DecoRNAi approach or CSA approach, and the Y-axis indicates the corresponding false positive rate.

GESS (genome-wide enrichment of seed sequence matches) is recently reported computational tool designed to identify off-targeted transcripts rather than to isolate and correct off-target phenotypes (8). We applied the GESS algorithm in an attempt to employ it for the latter. However, this method identified no off-target siRNA pools from either the H1155 toxicity screen or the selective autophagy screen. In stark contrast, this approach identified 23 807 off-target siRNA pools from the H1N1 cytopathogencity screen (Figure 6a–c). However, we anticipate that GESS's intended utility will dovetail with DecoRNAi, providing a mechanism to help identify gene cohorts that are responding to siRNAs responsible for seed-sequence driven phenotypes.

Two additional computational efforts designed to deflect spurious gene-level annotations from large-scale RNAi screens are ATARiS (Analytic Technique for Assessment of RNAi by Similarity) (12) and CSA (Common Seed Analysis) (13). ATARiS was developed to detect coherent behavior from multiple shRNAs targeting the same gene. While effective, the method is less generalizable outside of pooled shRNA screens and requires multi-sample RNAi screens (at least 10 samples in their publication). CSA, like DecoRNAi, detects correlated biological behavior of siRNAs that share the same seed sequence. However, CSA does not account for family-size bias with its statistical significance metric. Integration of statistical significance with the strength and direction of biological phenotypes is likely an important consideration for optimized detection of false positives (Supplementary Figure S4c–e). Furthermore, DecoRNAi quantifies seed-driven off-target effects by modeling the on-target effects and off-targets from all individual siRNA duplexes in the same gene pool, which is more efficient than looking at individual siRNA seed families separately. In support of these considerations, we found that the DecoRNAi corrected Z scores had a significantly better true positive rate than CSA corrections (Figure 6d).

A limitation of DecoRNAi is appropriate representation of seed families within a given screening collection to reach sufficient statistical power for detection of phenotypic associations. However, from the cumulative analysis of five different whole genome siRNA screens, we estimate that the DecoRNAi approach will cover ∼85% of the seed sequence families present in a typical commercial arrayed siRNA library (Supplementary Figure S4a and b). To facilitate automated application of DecoRNAi to siRNA and shRNA library screening efforts, we have embedded pre-computed seed family annotations for three commonly used commercial RNAi libraries (Dharmacon Library circa 2005, Dharmacon Library circa 2009 and Ambion Silencer). In addition, we proved a tool for automated generation of seed family annotation of user-specific siRNA or shRNA oligonucleotide collections (http://galaxy.qbrc.org/root?tool_id=sirna_offtarget). The tool is based on Galaxy open source framework, accepts phenotypic measures (such as *Z* scores) from primary screens as input, and allows iterative parameter choices for data analyses. All of the user-specified parameters are documented in detail, and the intermediate outputs are provided for transparent analysis (Figure 5, Supplementary User's Manual).

In summary, DecoRNAi is a computational tool that fills an important unmet need for the functional genomics research community as it enhances the return of rigorous biologically meaningful observations downstream of screening efforts that otherwise consume huge time and reagent resources by following bad leads, or by weeding them out using strictly empirical approaches. Substantial reduction of off-target rates was experimentally validated in five distinct biological screens across different genome-wide siRNA libraries. A public-access graphical user interface has been constructed to facilitate application of this algorithm by any investigator.

## SUPPLEMENTARY DATA

Supplementary Data are available at NAR Online.

SUPPLEMENTARY DATA
