# Supplementary material for: Computational detection and suppression of sequence-specific off-target phenotypes from whole genome RNAi screens
Source: Nucleic Acids Res. 2014 Jun 27;42(13):8214–22. doi: 10.1093/nar/gku306 (PMC4117740; doi:10.1093/nar/gku306)
Supplement: SUPPLEMENTARY DATA [file supp_42_13_8214__index.html]

Computational detection and suppression of sequence-specific off-target phenotypes from whole genome RNAi screens — SUPPLEMENTARY DATA 

# Computational detection and suppression of sequence-specific off-target phenotypes from whole genome RNAi screens

## SUPPLEMENTARY DATA

**Files in this Data Supplement:**

- SUPPLEMENTARY DATA
- SUPPLEMENTARY DATA
