# Supplementary material for: Computational detection and suppression of sequence-specific off-target phenotypes from whole genome RNAi screens
Source: Nucleic Acids Res. 2014 Jun 27;42(13):8214–22. doi: 10.1093/nar/gku306 (PMC4117740; doi:10.1093/nar/gku306)
Supplement: SUPPLEMENTARY DATA [file supp_gku306_nar-00120-n-2014-File008.pdf]

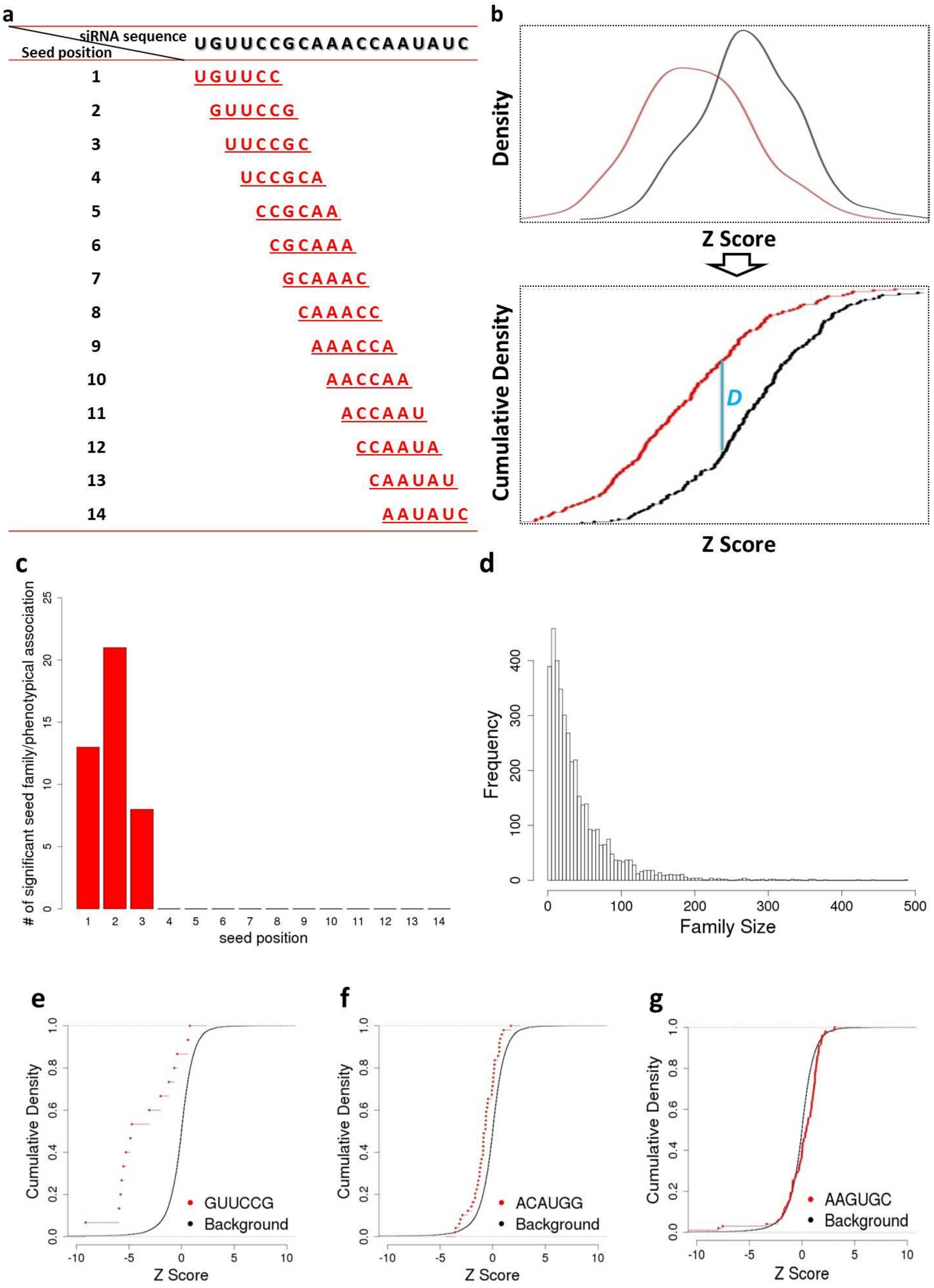

Supplementary figure S1. Detection of seed sequence enrichment in "active" siRNA pools from whole genome RNAi screens. (a) Illustration of 6-mer sliding window definitions of candidate seed regions. (b) Illustration of use of the KS-test as a scoring metric for detection of coherent behavior among siRNA pools with oligonucleotides containing a common seed sequence. KS-test

is a non-parametric approach and uses the maximum vertical deviation between the two distribution curves as the statistic D to examine if two distributions are statistically different. (c) Occurrence of seed sequence/phenotype associations within the H1155 toxicity screen for each seed region definition. (d) Frequency distribution of seed sequence family size as defined by nucleotides 2-7 in the Dharmacon siRNA library. (e-g) Examples of empirical distribution functions for three seed families of increasing size (15, 49 and 100, respectively) but with similar KS test p values (4.72×10^{-5}, 5.63×10^{-5} and 3.19×10^{-5}).

a

Seed family	Strength of seed-linked effect	Family size	Significance (P value)
GUUCCG	-3.77	15	4.72E-05
GUGUAC	-2.02	7	3.88E-03
UACUCC	-2.01	36	2.31E-04
ACAUGC	-1.72	21	2.87E-03
UUGCAG	-1.64	39	7.51E-04
CCCGCA	-1.32	11	9.46E-03
UAUGCC	-1.21	14	4.70E-04
UCCAGG	-1.17	44	5.24E-08
UCAGUU	-1.17	8	2.53E-03
UUCACC	-1.14	29	1.45E-04
UAUAGG	-1.05	69	2.74E-05
UAGGAG	-1.05	43	9.99E-04
ACUAGU	-1.04	31	1.16E-03

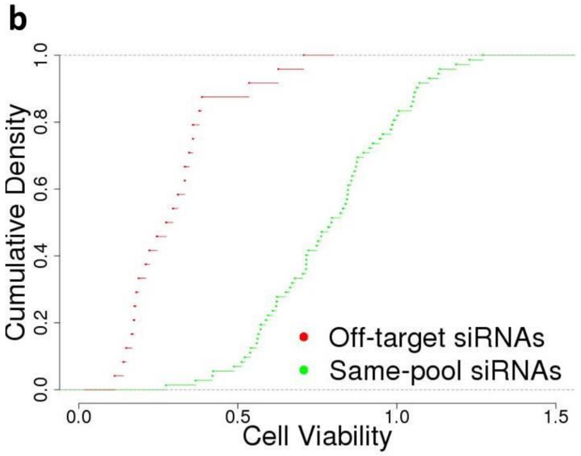

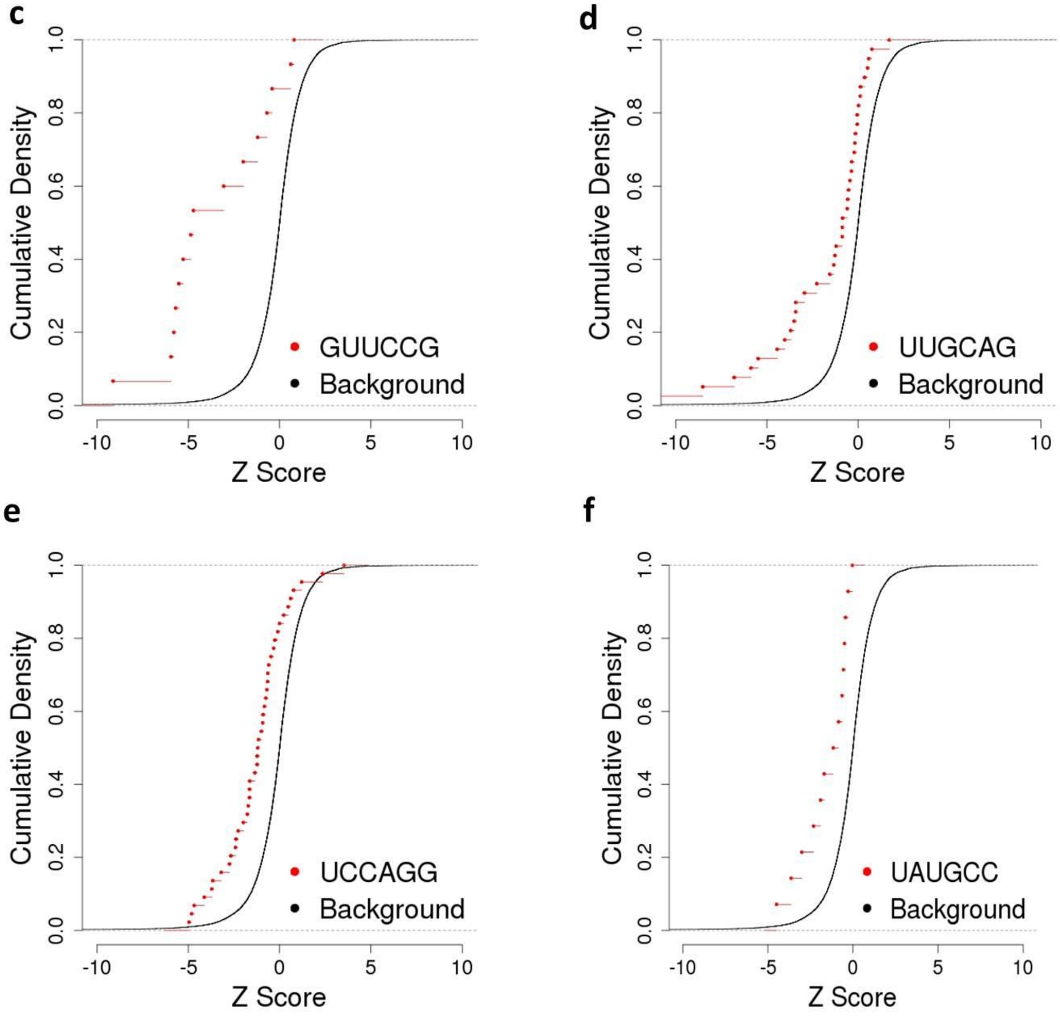

g

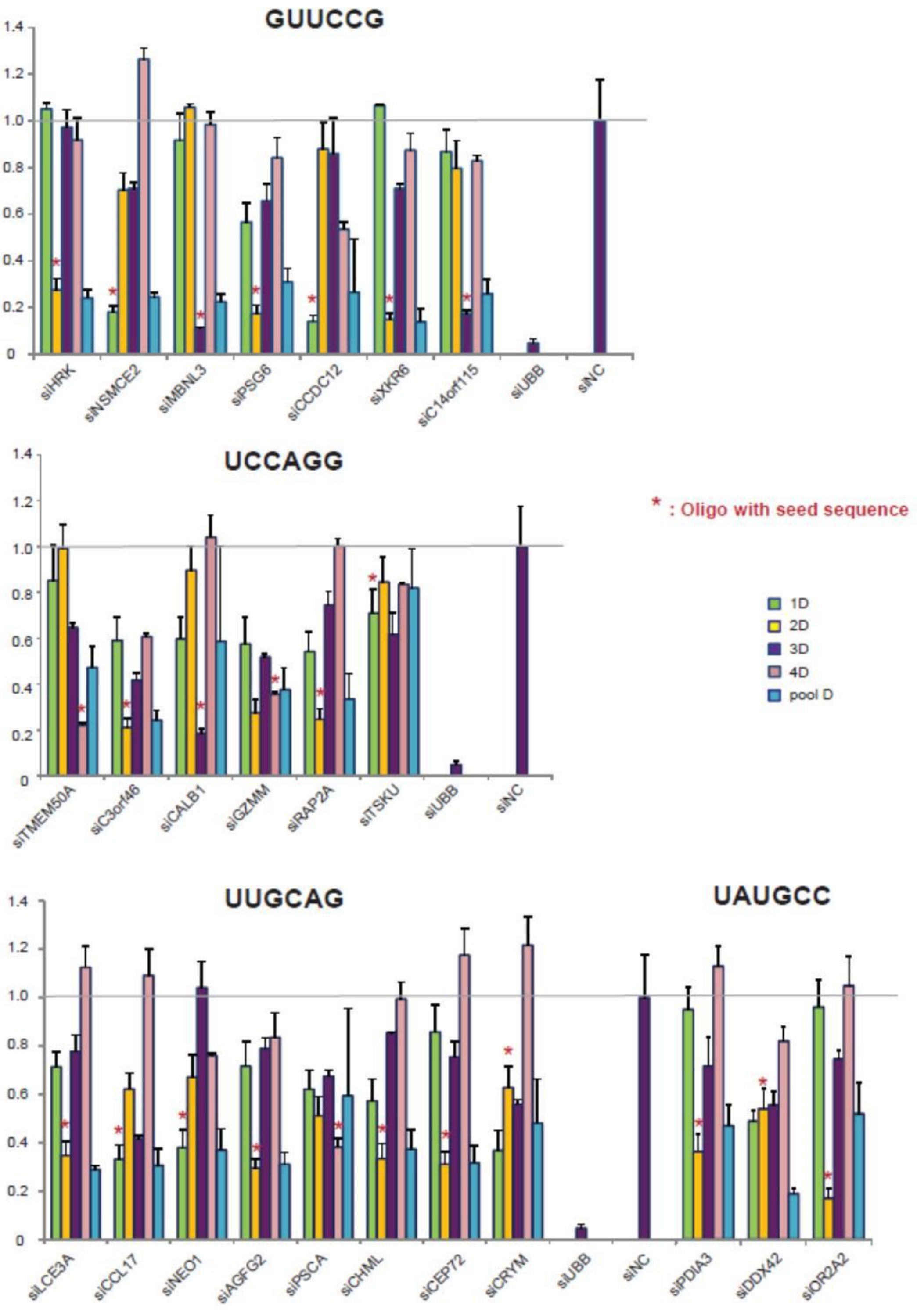

h

Seed family	miRNA family	MiRBase ID	Mature sequence
UAUGCC	miR-4633-5p	hsa-mir-4633-5p	A**UAUGCC**UGGCUAGCUCCUC

Supplementary figure S2. Experimental evaluation of DecoRNAi predictions in the H1155 toxicity screen. (a) A table of identified off-target seed families (strength less than -1 and p-value less than 0.01). GUUCCG (strength of seed- linked effect=-3.77, P value = 4.72×10-5) corresponds to the strongest predicted off-target effect among these 13 seeds. (b) The cumulative density distribution of cell viability values from siRNA duplex retests corresponding to 4 seed families with predicted off-target effects is shown. siRNA duplexes containing an oligonucleotide containing the predicted off-target seed sequence are indicated in red. siRNA duplexes targeting the same genes but lacking the predicted off-target seed sequence are indicated in green. (c) (d) (e) (f) The empirical distribution functions of the four seed families selected for evaluation are shown. (g) Individual siRNA duplex retests indicate that oligonucleotides containing the predicted off-target seed sequence are associated with the strongest phenotype. An astrix indicates oligos containing one of the four selected seed sequences. (h) Only the UAGUCC seed family corresponds to a known miRNA seed sequence.

a

Seed family	Strength of seed-linked effect	Family size	Significance (P value)
UGCGGU	-1.58	13	1.61E-03
UGGUAG	-1.39	22	7.06E-03
ACGUGG	-1.32	47	1.45E-03
UUCUGC	-1.13	34	5.67E-03
ACUGGG	-1.04	35	8.39E-04
AUCUGG	-1.02	139	6.32E-11
UGCUGU	-1.02	33	4.42E-03
UCAUGG	-1.00	31	3.47E-04
UUGGGU	1.01	32	4.03E-04
UAAUGC	1.01	25	5.27E-04
UACCCG	1.14	23	1.05E-03
UUGGUC	1.14	10	3.56E-04
UCCGUA	1.80	6	4.20E-03

b

Seed family	Strength of seed-linked effect	Family size	Significance (P value)
UGUUUU	-1.02	4	3.55E-03

c

Seed family	Strength of seed-linked effect	Family size	Significance (P value)
GCAUGG	-1.99	10	7.13E-03
CGUCAG	-1.43	6	9.83E-03
UAGGCA	-1.15	43	1.22E-04
AGGCAU	-1.15	24	7.82E-03
CCGAAU	-1.06	8	3.38E-03
UGUUGG	-1.04	35	9.97E-04

d

Seed family	Strength of seed-linked effect	Family size	Significance (P value)
ACAUGU	-1.29	43	4.42E-06
ACUACG	-1.37	7	7.39E-03
AGGUCG	-1.28	10	6.47E-04
AUGUCC	-1.02	16	6.48E-03
GUAGUU	-1.04	45	1.61E-06
UAGGUC	-1.46	63	1.28E-13
UAGUUG	-1.06	81	7.45E-08
UCGUAC	-1.34	10	3.64E-03
UCUGAC	-1.68	33	1.47E-04
UGCUCU	-1.20	10	1.68E-03

Supplementary figure S3. Application of DecoRNAi to additional RNAi screens with distinct biological contexts. (a) Identification and estimation of seed-sequence dependent effects from a genome-wide siRNA screen for modulators of H1N1 induced cell death in HBEC30 cells. A table of identified off-target seed families is shown. Annotation of all siRNA pools and their associated z-scores can be found in Supplemental Table 2. (b) Identification and estimation of seed-sequence dependent effects from a genome-wide siRNA screen for modulators of Wnt pathway signaling in HeLa cells dataset. DecoRNAi identifies only 1 phenotypical off-target effect seed family with marginally significant effects. A table ofidentified off-target seed families is shown. Annotation of all siRNA pools and their associated Z-scores from a luciferase reporter based scoring metric can be found in Supplemental Table 4. (c) Identification and estimation of seed-sequence dependent effects from a genome-wide siRNA screen for modulators of selective

autophagy in HeLa cells. A table of identified off-target seed families is shown. Annotation of all siRNA pools and their associated z-scores from an image-based scoring metric can be found in Supplemental Table 5. (d) Identification and estimation of seed-sequence dependent effects from a genome-wide siRNA toxicity screen in HCC4017 cells using an alternative siRNA library. A table of identified off-target seed families is shown. Annotation of all siRNA pools and their associated z-scores can be found in Supplemental Table 6.

a

Dataset	Family size range of off-target seed families
H1155	7~69
HBEC30	6~139
Image screen	6~43
Wnt pathway	4~8
HCC4017	7~81

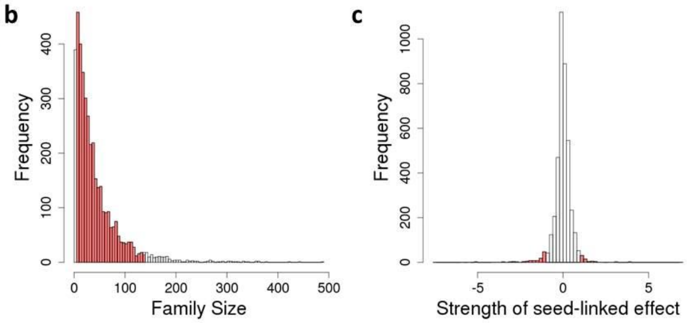

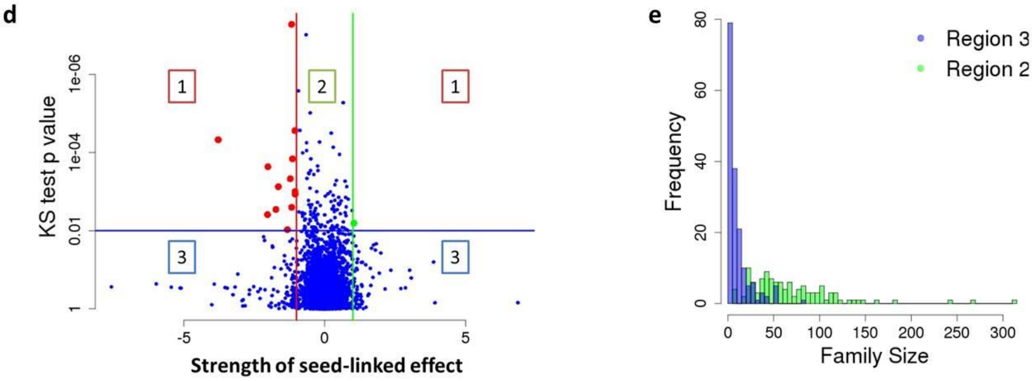

Supplementary figure S4. Coverage of seed family sizes detected within the DecoRNAi analysis and their relationship to LASSO coefficient threshold selection. (a) The family size ranges of predicted off- target seed families from all four datasets are indicated. (b) Of ~4000 seed families, seed family sizes ranging from 6 to 139 (red bars, 86% of total) were detectable within typical siRNA screens employing the Dharmacon library. (c) For these studies, the threshold selection for significant LASSO coefficients included 3% of each tail. This threshold is investigator tunable. (d) Classification of seed families based on LASSO coefficients and KS p values. Region 1: considered to be both biologically and statistically significant; Region 2: considered to be statistically significant but biologically insignificant; Region 3: considered to be biologically significant but statistically insignificant. (e) From the family size distribution, most seed families from region 3 have a family size of less than 6 and those from region 2 are enriched for the largest family sizes.

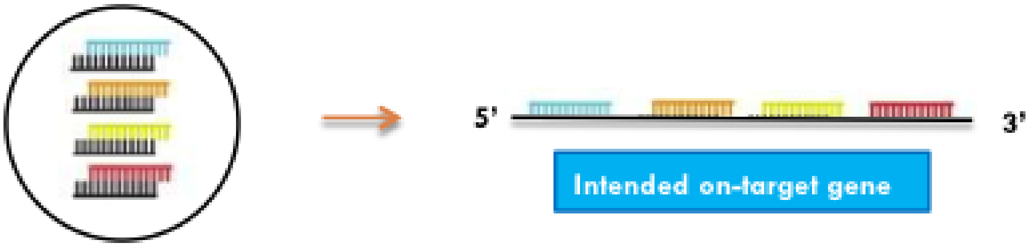

Supplementary figure S5. Pooled siRNAs targeting. The sequences of pooled siRNAs are different. However, by design, the siRNAs within the same pool should be targeting the same gene by perfectly matching on different location of the corresponding mRNAs as shown in figure. On the left panel, black line represents different locations/sequences from the same gene, the blue, brown, yellow and purple lines represent four different siRNAs targeting on the same gene. The right panel shows how these four siRNAs bind to the same gene. All the siRNA were designed perfectly matched to the targeting genes.
